# Needle-Free Bedside and Anthropometric Measures Show Weak Discrimination of an Adverse Blood-Biomarker Profile in Middle-Aged and Older Mexican Adults: An Internally Validated Machine-Learning Study

**DOI:** 10.3390/jcm15197454

**Published:** 2026-09-25

**Authors:** Exal Garcia-Carrillo, Eduardo Guzmán-Muñoz, Felipe Montalva-Valenzuela, Antonio Castillo-Paredes, Rodrigo Villaseca-Vicuña, Yeny Concha-Cisternas, Jose Jairo Narrea Vargas, Iván Molina-Marquez, Joaquín González-Aroca, Rodrigo Yáñez-Sepúlveda

**Affiliations:** 1 Department of Physical Activity Sciences, Faculty of Education Sciences, Universidad Católica del Maule, Talca 3480112, Chile; exal.garcia@gmail.com; 2 Department of Physical Activity Sciences, Universidad de Los Lagos, Osorno 5290000, Chile; 3 Escuela de Pedagogía en Educación Física, Facultad de Educación, Universidad Autónoma de Chile, Talca 3460000, Chile; 4 Escuela de Entrenador en Actividad Física y Deporte, Facultad de Ciencias Humanas, Universidad Bernardo O’Higgins, Santiago 8370993, Chile; 5 Grupo AFySE, Investigación en Actividad Física y Salud Escolar, Escuela de Pedagogía en Educación Física, Facultad de Educación, Universidad de Las Américas, Santiago 8370040, Chile; acastillop85@gmail.com; 6 School of Educational Sciences and Technology, Physical Education Pedagogy, Faculty of Education, Universidad Católica Silva Henríquez, Santiago 8280354, Chile; rvillaseca@ucsh.cl; 7 Escuela de Kinesiología, Facultad de Salud, Universidad Santo Tomás, Talca 3460000, Chile; yenyconchaci@santotomas.cl; 8 Vicerrectoría de Investigación e Innovación, Universidad Arturo Prat, Iquique 1100000, Chile; 9 Grupo de Investigación en Nutrición, Metabolismo y Ejercicio, Carrera de Nutrición y Dietética, Facultad de Ciencias de la Salud, Universidad Científica del Sur, Lima 15067, Peru; jnarrea@cientifica.edu.pe; 10 Pedagogía en Educación Física, Facultad de Educación, Universidad Adventista de Chile, Chillán 3780000, Chile; ivanmolina@unach.cl; 11 Escuela de Tecnología Médica, Facultad de Salud, Universidad del Alba, La Serena 1720236, Chile; joaquin.gonzalez@userena.cl; 12 Facultad de Educación y Humanidades, Escuela de Ciencias del Deporte, Universidad Andres Bello, Viña del Mar 2200055, Chile; rodrigo.yanez.s@unab.cl; 13 School of Medicine, Universidad Espíritu Santo, Samborondón 092301, Ecuador

**Keywords:** machine learning, aged, biomarkers, anthropometry, hand strength, gait speed, Mexico, C-reactive protein, vitamin D, cholesterol, HDL

## Abstract

**Background/Objectives**: Needle-free measures such as grip strength, gait speed, and anthropometry are attractive as potential substitutes for blood-based biomarkers in low-resource settings, yet whether they can predict an adverse blood-biomarker profile remains unknown. This study aimed to determine whether machine-learning algorithms can predict such a profile from a defined set of needle-free bedside and anthropometric measures in Mexican adults. **Methods**: Using the Mexican Health and Aging Study (MHAS) 2012 biomarker subsample (*n* = 2000; mean age 62.4 years; 59.8% women), we defined a blood-based outcome: two or more of four abnormal markers (high CRP, low HDL, high cholesterol, vitamin D deficiency). Needle-free predictors were age, sex, grip strength, gait speed, blood pressure, heart rate, pulse pressure, BMI, waist, hip, waist-to-height ratio, waist-to-hip ratio, knee height, and walking-aid use. A circularity audit confirmed zero overlap. Sixteen algorithms were compared by fivefold cross-validation. Logistic regression and LightGBM were trained on 80% of the data and evaluated on a held-out 20% test set, with bootstrap CIs, calibration, SHAP, decision-curve analysis, permutation tests of the complete pipeline, and continuous regression. Sensitivity analyses varied the outcome definition, class balancing, survey weighting, and medication-related proxies. **Results**: Cross-validated ROC AUC ranged 0.481 to 0.524. LightGBM achieved AUC 0.539 (95% CI 0.477–0.596), and logistic regression 0.584 (0.528–0.638) in the held-out set, but the median held-out AUC of logistic regression across 200 random partitions was 0.518. Permutation tests of the complete cross-validated pipelines did not separate performance from chance (*p* ≥ 0.38). Continuous regression yielded negative R^2^ values for all markers (−0.14 to −0.08). No marker, sex stratum (men AUC 0.457; women 0.54), or sensitivity analysis reached useful discrimination (all AUC ≤ 0.58), and no model showed net benefit. SHAP importance was diffuse, with grip and gait ranked below all anthropometric and vital-sign features. **Conclusions**: In this sample, the evaluated bedside and anthropometric measures carried at most weak information about the predefined blood-biomarker profile, well below the level required for clinical use. They should not replace laboratory measurement of these biomarkers when biochemical assessment is clinically indicated.

## 1. Introduction

Populations are aging faster than health systems can adapt, and the burden of that transition falls hardest where laboratory infrastructure is thinnest [1,2]. Biological aging is reflected in both clinically observable characteristics and circulating biomarkers. Bedside measures such as grip strength, gait speed, and anthropometry provide accessible information on physical function and body composition, whereas circulating biomarkers characterize biological processes such as inflammation, lipid metabolism, and micronutrient status. Both domains are associated with adverse health outcomes [3,4,5], and both have been distilled into elegant clocks, including epigenetic, phenotypic, and allostatic measures, that estimate how fast a body is aging [6,7,8]. The visible language is cheap. Grip strength predicts cardiovascular death more strongly than systolic pressure across 17 countries [9]. It forecasts mortality and disease incidence in half a million UK Biobank participants [10]. Gait speed and the short physical performance battery stratify survival in older adults [11,12]. Low strength anchors the modern definitions of sarcopenia and frailty [13,14,15]. Skeletal muscle is increasingly recognized as an endocrine organ whose secretory activity interacts with adipose tissue, the liver, and the vascular system [16], and physical performance tracks with inflammation, anemia, and adiposity [17,18,19,20].

From these true associations, a hopeful inference has spread: if the visible tracks the invisible, perhaps the visible can stand in for it. This would take the form of a needle-free triage that flags who carries a hostile blood profile (elevated CRP, low HDL, high cholesterol, deficient 25[OH]D) without the cost, cold chain, phlebotomy, and assay standardization that blood work demands [21,22,23,24,25,26,27]. The inference is attractive precisely where it would matter most: in older adults, in Latin America, in settings where a laboratory is a day’s travel away [28,29,30,31,32,33,34]. This inference is attractive, but it remains untested at scale. The Mexican Health and Aging Study (MHAS) is a nationally representative panel of adults ≥50 years [35], embedded in a population with high and rising cardiometabolic risk [36,37,38] and documented micronutrient and sarcopenia burden across the region [39,40]. Mexico therefore provides a clinically and public-health relevant setting in which to test this hypothesis. A rigorous test in this setting is informative for health systems that consider needle-free alternatives to blood testing [41,42].

Modern machine learning has been offered as the bridge that would finally let cheap inputs reconstruct expensive outputs [43,44]. The promise is that flexible, non-linear algorithms can extract subtle patterns that traditional regression models miss, potentially bridging the gap between visible and invisible phenotypes. Yet the same literature warns that flexible models rarely beat logistic regression [45], that they are frequently miscalibrated [46], that convenient proxies encode bias [47], and that model complexity does not necessarily translate into stronger predictive information [48,49]. A systematic review of 71 clinical prediction studies found no performance benefit of machine learning over logistic regression [45]. When ML models do appear to excel, this superiority often dissolves under rigorous validation [50], revealing overfitting, data leakage, or optimistic bias [51]. Furthermore, even well-performing models frequently fail the calibration test [46], making their probability estimates clinically unusable. This pattern has led to calls for more rigorous, skeptical evaluation of ML in clinical contexts [52,53,54].

Our study was designed to follow current standards for trustworthy prediction. We adopted the TRIPOD+AI guidelines [52,53], including mandatory elements often missing from the machine-learning literature: a circularity audit, planned before modeling, to confirm that no predictor is an algebraic component or definitional element of any outcome marker; sample-size justification based on contemporary minimums for stable estimation and validation [55,56]; leakage-controlled preprocessing where all imputation and oversampling are performed inside training folds only; calibration assessment with isotonic recalibration [46]; decision-curve analysis to evaluate net clinical benefit rather than statistical significance alone [57,58]; label-permutation tests of the complete pipeline to establish the null distribution; and continuous-outcome regression for each biomarker, which cannot be blamed on dichotomization. We also decided before analysis to report the result regardless of its direction, recognizing that null results from rigorous testing are systematically suppressed in the literature [51], yet provide essential scientific anchoring. The analysis plan was not preregistered or deposited in a public registry. Throughout this article, “planned” and “a priori” denote decisions the authors made before fitting the models. They lack the independent verifiability of a formal preregistration. This study therefore tested, using a broad set of machine-learning algorithms, whether a defined set of needle-free measures can predict an adverse blood-biomarker profile in the MHAS 2012 biomarker subsample.

We therefore posed a single, falsifiable question: Can any of a broad set of machine-learning algorithms predict an adverse blood-biomarker profile from 15 needle-free bedside and anthropometric measures in Mexican adults from the MHAS 2012 biomarker subsample? We address it with the standard components of prediction-model evaluation. This includes discrimination, calibration, decision-curve analysis, permutation testing, and continuous-outcome regression, all under TRIPOD+AI [52,53,54]. The question concerns prediction for individuals. It does not test whether physical and biochemical measures are associated at the population level, which is a different question [59].

## 2. Materials and Methods

### 2.1. Study Design, Data Source, and Participants

MHAS is a prospective, nationally representative panel study of Mexican adults ≥50 years with a biomarker substudy collected in 2012 [35]. In 2012, venous blood was collected from a random subsample of MHAS respondents [60,61]. We analyzed the section-L biomarker file (N = 2087). After restricting to participants with a computable outcome, the analytic sample was *n* = 2000 (mean age 62.4 ± 10.6 years; 59.8% women). MHAS enrolls adults aged ≥50 years together with their spouses or partners regardless of age; 143 participants (7.2%) were younger than 50 years and were retained in the main analysis. Blood samples were drawn between October and December 2012. The file includes the MHAS 2012 individual sampling weight, which was zero for 174 participants. Because the aim was to evaluate individual-level prediction within the analytic sample, the main analyses were unweighted, and survey-weighted estimates are reported as a sensitivity analysis (Section 2.6). The unweighted estimates therefore describe the biomarker subsample and should not be read as population prevalence or population-level performance. Reporting follows TRIPOD+AI [52,53], and risk of bias was self-appraised against PROBAST [62].

### 2.2. Outcome: The Blood-Biomarker Profile

The outcome was deliberately confined to venous blood. Four clinically standard abnormalities were defined a priori: high-risk systemic inflammation (CRP category > 3 mg/L) [26]; low HDL cholesterol (<40 mg/dL) [24]; high total cholesterol (≥240 mg/dL) [25]; and vitamin D deficiency (25[OH]D < 20 ng/mL) [21,22]. The primary outcome (i.e., adverse profile) was the presence of ≥2 of these four (prevalence 43%). Component prevalence was CRP 43%, low HDL 47%, high cholesterol 16%, and vitamin D deficiency 32%. Each abnormality was also modeled separately. We do not regard this composite as a validated biological phenotype. The four markers reflect distinct processes, and different pairs, such as high CRP with vitamin D deficiency or low HDL with high total cholesterol, qualify identically. The composite is a pragmatic operational target that mirrors the intended use of a needle-free triage tool: to flag individuals in whom blood testing would reveal more than one common, clinically actionable abnormality. Equal weighting follows the count-based convention of allostatic-load scores, which sum dichotomized markers when no external criterion is available to weight them [8,63]. The ≥2 threshold was chosen to capture co-occurring abnormalities rather than a single, possibly transient, value. It is also the only cutoff point that divides the sample into groups of comparable size (≥1 marker, 83.9%; ≥3 markers, 10.8%). Because the composite reflects a construct decision, we assessed the robustness of the predictive result separately, using alternative thresholds and composites, each marker alone, the ordinal count of abnormal markers, and continuous marker values (Section 2.5 and Section 2.6). Participants were not excluded on the basis of lipid-lowering or anti-inflammatory medication. Medication use is not recorded in the biomarker file analyzed here, so treated participants could not be identified. Because treatment can shift measured marker values systematically rather than add random noise, we performed proxy-based sensitivity analyses (Section 2.6) and discuss the implications in Section 4.

### 2.3. Predictors: The Visible (Needle-Free) Layer

Fifteen predictors were obtainable without any blood draw: age; sex; right-hand grip strength (best of two trials); 4 m gait speed (mean of two trials); resting systolic and diastolic blood pressure and heart rate (mean of two seated measurements); pulse pressure; body-mass index; waist and hip circumference; waist-to-height and waist-to-hip ratios [20]; knee height; and walking-aid use [12,13,15]. Grip and gait were operationalized as per consensus protocols [13,15].

### 2.4. Circularity Audit

This audit was mandatory and planned before modeling. No predictor is an algebraic component, coding artifact, or device-derived transform of any outcome marker. The outcome is exclusively blood-based, and predictors are exclusively physical, anthropometric, or vital-sign based. Adiposity (waist, BMI) is biologically correlated with lipids and inflammation. That correlation is the legitimate signal under test, not leakage, but it is not a definitional element of CRP, HDL, cholesterol, or 25(OH)D. Metabolic syndrome, whose waist criterion would introduce circularity with anthropometric predictors, was therefore excluded as an outcome by design.

### 2.5. Modeling, Validation, and Reproducibility

Sixteen algorithms (Figure 1 spanning linear, kernel, neighborhood, tree-ensemble, boosting, and neural families were compared by stratified 5-fold cross-validation with ROC AUC as the primary metric [45]. All preprocessing (median imputation, standardization) and SMOTE oversampling were fit inside training folds only, preventing leakage. SMOTE was applied uniformly to every binary outcome, including low-prevalence secondary outcomes (high total cholesterol, 16%). The primary outcome (43%) is not severely imbalanced, and resampling can distort predicted probabilities [64]; the main analyses were therefore repeated without SMOTE (Section 2.6). Two models were then trained on a stratified 80% partition and evaluated once on the held-out 20% test set, with 800-sample bootstrap confidence intervals: logistic regression, as the mandatory baseline, and LightGBM, as the representative flexible gradient-boosted model. LightGBM was not selected for having the highest cross-validated AUC. The decision tree ranked first (0.524; Figure 1), and all between-algorithm differences were small relative to resampling variability. To show that this choice did not drive the conclusion, we also applied a data-driven rule restricted to the training partition. The 16 algorithms were ranked by 5-fold cross-validated AUC within the training 80%, and the top-ranked algorithm was evaluated once on the held-out 20%. The held-out partition was not used for model selection or hyperparameter tuning. Probabilities were isotonically recalibrated; the calibration slope and intercept and the Brier score are reported alongside discrimination [46,54]. Clinical usefulness was assessed by decision-curve analysis [57,58]. To guard against over-interpretation of small effects, we added three analyses. First, label-permutation tests (300 permutations) reran the complete cross-validated pipeline, including imputation, scaling, SMOTE, and model fitting, on each permuted outcome for logistic regression and for LightGBM. The test statistic was the pooled out-of-fold AUC, and *p* = (1 + number of permuted AUCs ≥ observed AUC)/301. The same test was applied to the fixed-partition held-out logistic-regression procedure. Second, the held-out analysis of both models was repeated over 200 random stratified 80/20 partitions, to quantify how much a single-partition estimate depends on the split [65]. Third, continuous-outcome regression (cross-validated R^2^) was fitted for each biomarker, which cannot be blamed on dichotomization. Sample size exceeded contemporary minimums for stable estimation and validation [55,56]. Feature attributions used SHAP TreeExplainer and are interpreted as associational, not causal [49]. The random seed was set to 42 for all stochastic steps; analyses used Python (v3.11), with scikit-learn (v1.8), LightGBM (v4.3.0), XGBoost (v2.0.3), CatBoost (v1.2.5), imbalanced-learn (v0.12.0), and SHAP (v0.45.0). Because negative predictive findings are as susceptible to bias as positive ones, we followed guidance on reporting completeness and reproducibility throughout [50,51,66,67,68,69]. For gradient-boosted trees (LightGBM, XGBoost, CatBoost), hyperparameter optimization was performed using a randomized search approach within the training folds, focusing on learning rate, tree depth, and regularization parameters to reduce the chance that poor performance reflected suboptimal configuration.

### 2.6. Sensitivity and Supplementary Analyses

The analyses introduced during peer review, namely the permutation tests and repeated partitions described above, the training-partition selection rule, and the sensitivity analyses below, are post hoc. They used a reimplementation of the pipeline (Python, scikit-learn 1.8, LightGBM, imbalanced-learn) that reproduces the cross-validated logistic-regression AUC in Figure 1 (0.507). For the held-out logistic-regression analyses, the configuration without SMOTE was used because it reproduced the held-out estimate in Table 1 (AUC 0.585 vs. 0.584; with SMOTE, 0.571). Each sensitivity analysis used logistic regression and LightGBM with default hyperparameters, stratified 5-fold cross-validation (seed 42), the pooled out-of-fold AUC with 1000-sample bootstrap 95% CIs, and, as a stability check, the mean AUC over 10 repetitions of cross-validation with different seeds.

Six sets of sensitivity analyses were performed. (a) Alternative outcome definitions: ≥1 and ≥3 abnormal markers; a cardiometabolic composite of ≥2 of CRP, HDL, and total cholesterol, excluding vitamin D; any lipid abnormality; and the ordinal count of abnormal markers (0–4), modeled as a continuous outcome. (b) The pipeline without SMOTE, with calibration slope, calibration-in-the-large, and Brier score. (c) Survey weighting, using the MHAS individual weight in model training and in a weighted AUC, in the 1826 participants with a positive weight. (d) Medication-related proxies. Because medication use is not recorded, we repeated the analyses in participants with point-of-care HbA1c < 6.5%, in whom diabetes, and therefore guideline-indicated lipid-lowering therapy, is less likely. We also repeated them in participants without any health insurance, who have less access to prescribed pharmacotherapy. HbA1c was used only to define the stratum, never as a predictor. (e) Restriction to participants aged ≥50 years. (f) An extended needle-free predictor set, adding day of blood draw, locality size, health-insurance affiliation, and new versus follow-up cohort, which are available without a blood draw.

## 3. Results

### 3.1. Cohort and Prevalence of the Adverse Blood-Biomarker Profile

Among 2000 participants, 43% met the criteria for the adverse blood-biomarker profile, with high CRP and low HDL cholesterol the most prevalent abnormalities (Figure 2). Predictor missingness was below 2.5% for all variables. With the MHAS individual sampling weight (*n* = 1826 with a positive weight), the weighted prevalence of the adverse profile was 45%.

### 3.2. No Algorithm Reached Useful Discrimination

Across the 16-algorithm sweep, cross-validated discrimination clustered tightly around chance (ROC AUC 0.481–0.524; Figure 1). Neither flexible gradient boosting nor the mandatory logistic-regression baseline approached the conventional useful threshold of 0.70 [45]. Predictive performance was similarly poor across linear, kernel-based, ensemble, boosting, and neural-network approaches. Between-algorithm differences (range 0.481–0.524) were small relative to resampling variability, so the ranking in Figure 1 does not identify a superior algorithm.

### 3.3. Held-Out Performance, Permutation Tests, and Continuous Outcomes

On the held-out test set, LightGBM reached AUC 0.539 (95% CI 0.477–0.596), whose confidence interval crossed 0.50, while logistic regression achieved 0.584 (0.528–0.638); both models showed poor calibration (Figure 3A,B). The logistic-regression interval excludes 0.50, and a permutation test of this fixed-partition procedure gave p = 0.007. In this partition therefore, there was statistical evidence of above-chance discrimination. The estimate did not generalize across partitions. Over 200 random 80/20 splits, the median held-out AUC of logistic regression was 0.518 (2.5th–97.5th percentile 0.473–0.567), and only two splits (1%) reached 0.584; for LightGBM, the median was 0.507 (0.453–0.555). When the complete cross-validated pipeline was rerun on 300 label permutations, observed performance was not separable from the permutation null for logistic regression (AUC 0.507; null mean 0.500; *p* = 0.389) or LightGBM (AUC 0.507; null mean 0.502; *p* = 0.379; Figure 3C). The data-driven rule applied within the training partition selected k-nearest neighbors (k-NN; training cross-validated AUC 0.510), which reached a held-out AUC of 0.472; the decision tree, first in Figure 1, reached 0.500. The single above-chance estimate thus reflects a favorable partition. At most, it indicates weak information, far below the AUC of 0.70 conventionally regarded as useful [45]. Critically, continuous regression (immune to any loss from dichotomization) produced negative cross-validated R^2^ for every marker (log-CRP −0.13; HDL −0.13; total cholesterol −0.14; 25[OH]D −0.08; Figure 3D), i.e., the models predicted worse than the sample mean.

### 3.4. SHAP Feature Attribution

SHAP attributions on the flexible model were thinly and diffusely spread across correlated anthropometric and vital-sign features, with grip strength and gait speed (the very measures the surrogate hypothesis relies upon) ranked below all anthropometric and vital-sign features, above only sex and walking-aid use (Figure 4). Given the poor held-out discrimination, these feature attributions should be interpreted cautiously and should not be considered evidence of stable or generalizable predictive relationships [48,49]. Beyond global importance, we examined SHAP dependence plots for key variables (e.g., age vs. BMI) to detect potential non-linear interactions; however, no consistent interaction structure emerged that exceeded the noise floor.

### 3.5. No Clinical Utility and No Rescuing Subgroup

Decision-curve analysis showed the model offered no robust net benefit over the default “treat-all” and “treat-none” strategies across the clinically relevant threshold range (Figure 5A) [57]. Per-marker discrimination remained near chance (AUC 0.49–0.56; Figure 5B), and sex stratification offered no rescue (men AUC 0.457, *n* = 805; women AUC 0.54, *n* = 1195; Figure 5C).

### 3.6. Sensitivity Analyses

No sensitivity analysis produced clinically useful discrimination (Table 2). Across the 12 specifications in Table 2, cross-validated AUCs ranged from 0.489 to 0.573, and repeated cross-validation changed them by at most 0.02. Alternative outcome definitions did not reveal a signal hidden by the ≥2 composite: AUCs were 0.52–0.53 for ≥1 abnormal marker, 0.52 for ≥3, 0.50–0.52 for the cardiometabolic composite without vitamin D, and 0.50–0.51 for any lipid abnormality. The ordinal count of abnormal markers was not predicted either (cross-validated R^2^ −0.004 for linear regression and −0.128 for LightGBM; Spearman ρ 0.04 and 0.03).

Removing SMOTE left discrimination unchanged (cross-validated AUC 0.510 vs. 0.507 for logistic regression and 0.506 vs. 0.507 for LightGBM) and improved calibration in the large. The mean predicted probability of logistic regression fell from 0.498 to 0.428, matching the observed prevalence of 0.428, and its calibration intercept moved from −0.29 to 0.00. Calibration slopes remained far from 1 in both configurations (logistic regression 0.11 and 0.16; LightGBM 0.03 and 0.02). This pattern reflects the absence of a usable signal rather than the resampling step.

Survey weighting did not change the conclusion. In the 1826 participants with a positive weight, weighted AUCs were 0.537 (95% CI 0.477–0.587) for logistic regression and 0.552 (0.506–0.597) for LightGBM, compared with unweighted values of 0.533 and 0.518 in the same participants. In the medication-proxy analyses, prediction did not improve among participants with HbA1c < 6.5% (primary outcome, 0.505 and 0.514; low HDL, 0.481 and 0.508; high total cholesterol, 0.499 and 0.549). Among the 349 uninsured participants, logistic regression reached 0.573 (0.509–0.632), whereas LightGBM reached 0.539 (0.480–0.600). The high-cholesterol estimates in this stratum fell well below 0.50 (0.461 and 0.380), which shows that estimates in small strata are unstable in both directions. Restricting the sample to participants aged ≥50 years gave AUCs of 0.489 and 0.492.

Adding needle-free contextual information did not improve prediction of the primary outcome (0.507 and 0.502). For vitamin D deficiency, it raised the logistic-regression AUC from 0.562 to 0.579 (95% CI 0.554–0.605). This small gain is consistent with a contribution of contextual determinants of vitamin D status, but discrimination remained far from useful. Because all blood samples were drawn between October and December 2012, seasonal contrast within the sample was limited.

### 3.7. Summary of Performance

To provide a consolidated overview of the predictive landscape, the performance metrics across all evaluation frameworks are synthesized in Table 1. This summary confirms the convergence of evidence from the initial 16-algorithm sweep, the held-out validation, and the secondary analyses (permutation, continuous regression, sex stratification, and sensitivity analyses). Regardless of the modeling approach or the clinical threshold applied, the evaluated needle-free measures showed at most weak discrimination of the adverse blood-biomarker profile. Cross-validated, permutation-tested, and continuous-outcome results were compatible with chance. The held-out logistic-regression estimate exceeded chance in its partition, but did not generalize across partitions, and no analysis approached clinically useful discrimination (Table 1 and Table 2).

## 4. Discussion

The main finding of this study was that the evaluated needle-free bedside and anthropometric measures showed limited ability to predict the predefined adverse blood-biomarker profile. Across multiple machine-learning algorithms and complementary validation approaches, discrimination remained poor, calibration was suboptimal, continuous-outcome prediction yielded negative cross-validated R^2^ values, and decision-curve analysis showed no meaningful clinical net benefit. These convergent findings suggest that the evaluated needle-free measures alone are insufficient for clinically useful prediction of this blood-biomarker profile. This conclusion concerns individual-level prediction from the measures evaluated here. It does not imply that physical characteristics and these biomarkers are biologically unrelated, because an association can be statistically robust and yet explain too little between-person variation to classify individuals [59].

Grip strength, gait speed, and adiposity are established prognostic markers [9,10,11,20], but a marker of future risk is not the same object as a snapshot of present biochemistry. Muscle and vessel and blood share final common pathways over years [4,5,16]; they do not move in lockstep in a single cross section. CRP is dominated by acute and stochastic processes; HDL and cholesterol are shaped by genetics, diet, and pharmacotherapy [24,25,38]; 25(OH)D reflects sunlight, intake, and season [21,39]. None of these is legible in how hard a hand squeezes today. That the visible and the invisible are both consequences of aging does not make one a readout of the other, a distinction the surrogate literature has too often elided [3]. These distinct determinants offer a plausible explanation for the weak cross-sectional prediction observed in this sample, but our design cannot show that physical and biochemical aging are biologically decoupled, let alone universally so. Population-level work illustrates the distinction. Physical activity, a non-invasive behavioral measure, has been associated with a blood-derived epigenetic aging indicator (DNAm PhenoAge), with β2-microglobulin examined as a potential link [70]. Associations of this kind describe average differences between groups; our results address the separate question of whether needle-free measures can classify individuals.

Pharmacotherapy may weaken prediction in a way that is not random. Participants whose adiposity or blood pressure signal an adverse profile may be more often treated with statins or anti-inflammatory drugs. Treatment would then lower CRP and total cholesterol precisely in those flagged by the predictors [27], systematically weakening the observable predictor–outcome relationship. Medication use is not recorded in the biomarker file, so we could not adjust for it directly. The proxy analyses offer partial reassurance. Prediction did not improve among participants with HbA1c < 6.5%. Among the 349 uninsured participants, the only increase, for logistic regression, was small, imprecise, and not replicated by LightGBM; both groups are less likely to receive lipid-lowering treatment. Neither proxy identifies untreated individuals, however. Our results therefore describe prediction of measured, partly treated marker values. This is the quantity a triage tool would face in practice, but it is not evidence about untreated biology.

Our result is consistent with and extends three strands of methodological evidence. First, flexible algorithms did not beat logistic regression; here neither reached useful discrimination, echoing the systematic finding that machine learning confers no automatic advantage [45]. Second, the models were poorly calibrated even where discrimination was near chance, a reminder that calibration is the Achilles heel of predictive analytics [46,54]. Third, decision-curve analysis showed that acting on the model would not improve on default strategies across the thresholds examined [57,58]. Where much of the prediction literature is optimistically biased and under-validated [50,51,69], the value of a carefully validated negative result is to show that this particular set of measures should not replace these blood tests.

From an equity perspective, needle-free measures may appear attractive in settings where access to laboratory testing is limited, particularly among underserved aging populations [29,33,41,42]. However, the poor predictive performance observed in this study suggests that the evaluated measures should not be used as substitutes for blood-based assessment of the biomarkers examined. Reliance on inadequately performing proxies may lead to misclassification, including missed confirmatory assessment among individuals with abnormal biomarker profiles and unnecessary referrals among those without such abnormalities. Because proxy variables selected primarily for convenience or cost may contribute to inequities and algorithmic harm [47], strategies aimed at improving access to laboratory testing and assay standardization [22,26] may be preferable to replacing biochemical measurements with needle-free measures. Bedside and anthropometric assessments should therefore be regarded as complementary tools for functional and prognostic evaluation [9,11,13], rather than as substitutes for blood-based biochemical assessment. Our study did not compare strategies for addressing unequal access, such as expanded laboratory capacity, point-of-care or dried-blood-spot testing, or other non-invasive approaches. It therefore cannot show which strategy is optimal or cost-effective, or that needle-free approaches other than those evaluated here would fail.

### Strengths and Limitations

The main strengths of this study include the use of a large population-based sample, a separation between predictors and outcomes defined before modeling, evaluation of a broad range of machine-learning algorithms alongside a logistic-regression baseline, leakage-controlled preprocessing, and multiple complementary approaches to model evaluation. The inclusion of permutation testing, continuous-outcome regression, calibration assessment, decision-curve analysis, and sex-stratified analyses provides convergent information regarding predictive performance. Sensitivity analyses of the outcome definition, class balancing, survey weighting, medication-related proxies, and predictor set gave consistent results.

Findings are cross-sectional and specific to this 2012 wave and marker panel; grip was right-hand only at scale, and left-hand asymmetry could not be examined; the composite could mask a narrow signal for an unmeasured marker. Validation was internal only: cross-validation and a single held-out partition drawn from the same MHAS 2012 sample, supplemented by repeated random partitions; no external cohort was examined. The analysis plan was not preregistered, and the sensitivity analyses were added during peer review. Medication use was unavailable, so its influence could be examined only through proxies. The main analyses were unweighted, and the biomarker subsample may not reproduce the population distribution of the full MHAS sample. Finally, the 15 predictors do not exhaust needle-free information: other clinical, behavioral, environmental, or contextual measures could carry additional information. External validation in other cohorts (e.g., NHANES, ELSA, SHARE) and against continuous aging clocks [6,7] would test the generality of the null, following minimum-sample guidance for validation [55,56]. While the data originate from the 2012 MHAS wave, this remains one of the largest biomarker panels available for older Mexican adults. Whether more recent cohorts, other populations, or other measures would show stronger prediction is an empirical question that requires formal replication.

## 5. Conclusions

This study found that the evaluated needle-free bedside and anthropometric measures provided at most weak discrimination of the predefined adverse blood-biomarker profile in the MHAS 2012 biomarker subsample of Mexican adults, most of whom were aged 50 years and older. Across conventional regression and multiple machine-learning approaches, predictive discrimination remained poor, and complementary analyses, including sensitivity analyses of the outcome definition, class balancing, survey weighting, and medication-related proxies, provided no evidence of clinically useful model performance.

Although bedside and anthropometric measures remain valuable for assessing physical function, frailty, and prognosis, the measures evaluated in this study should not be considered substitutes for laboratory assessment of CRP, HDL cholesterol, total cholesterol, or vitamin D status. Their use alone for identifying individuals with the predefined adverse biomarker profile would result in substantial uncertainty and potential misclassification.

These findings support continued access to laboratory testing when biochemical assessment is clinically indicated and caution against replacing blood-based measurements with the needle-free proxies evaluated in this study. Future research should determine whether alternative combinations of non-invasive measures, additional clinical information, or external validation in other populations can improve prediction of specific circulating biomarkers. The present findings do not establish that other needle-free approaches would fail or which strategy best addresses unequal access to laboratory testing.

## Figures and Tables

**Figure 1:**
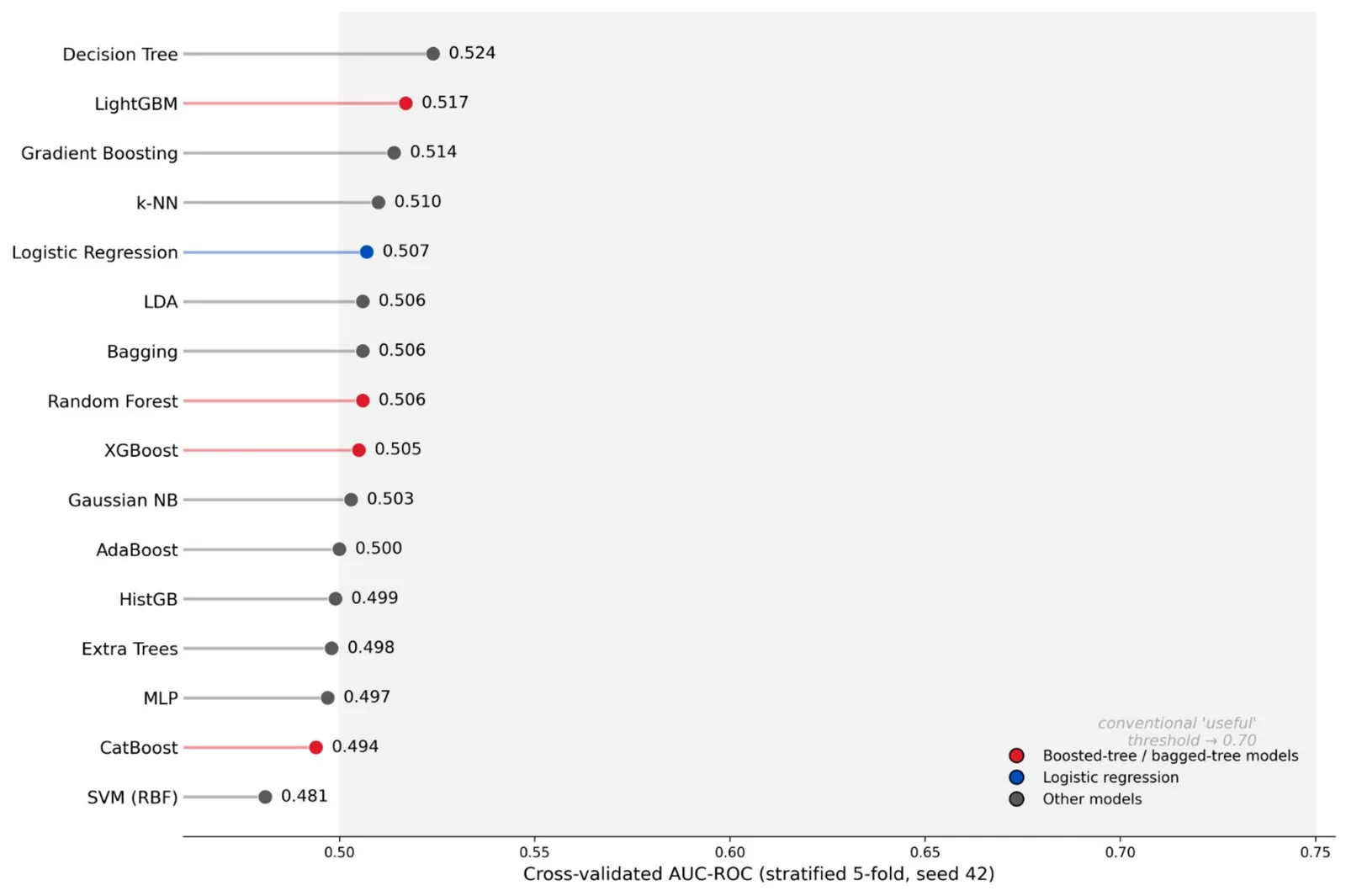
Sixteen-algorithm comparison of discrimination for the adverse blood profile from needle-free measures (stratified 5-fold cross-validation, seed 42). Every model clusters near chance, and between-model differences are within resampling variability; the shaded region marks the conventional “useful” threshold (0.70).

**Figure 2:**
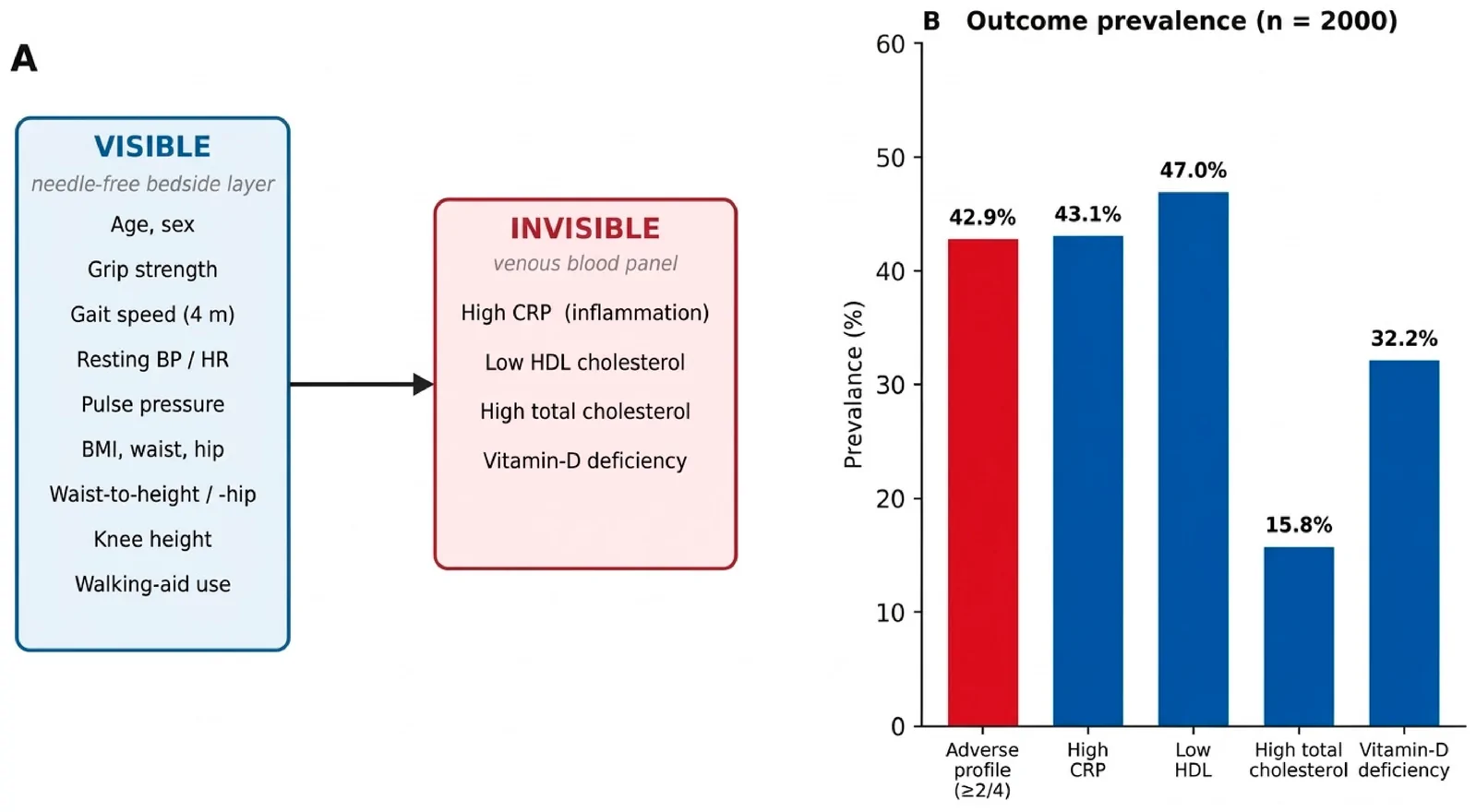
Study framework and blood-biomarker burden (MHAS 2012, *n* = 2000). (**A**) The conceptual separation between the needle-free “visible” layer and the blood-based “invisible” layer, with the machine-learning question posed across the divide. (**B**) Prevalence of the adverse profile (≥2 abnormal markers) and its four components.

**Figure 3:**
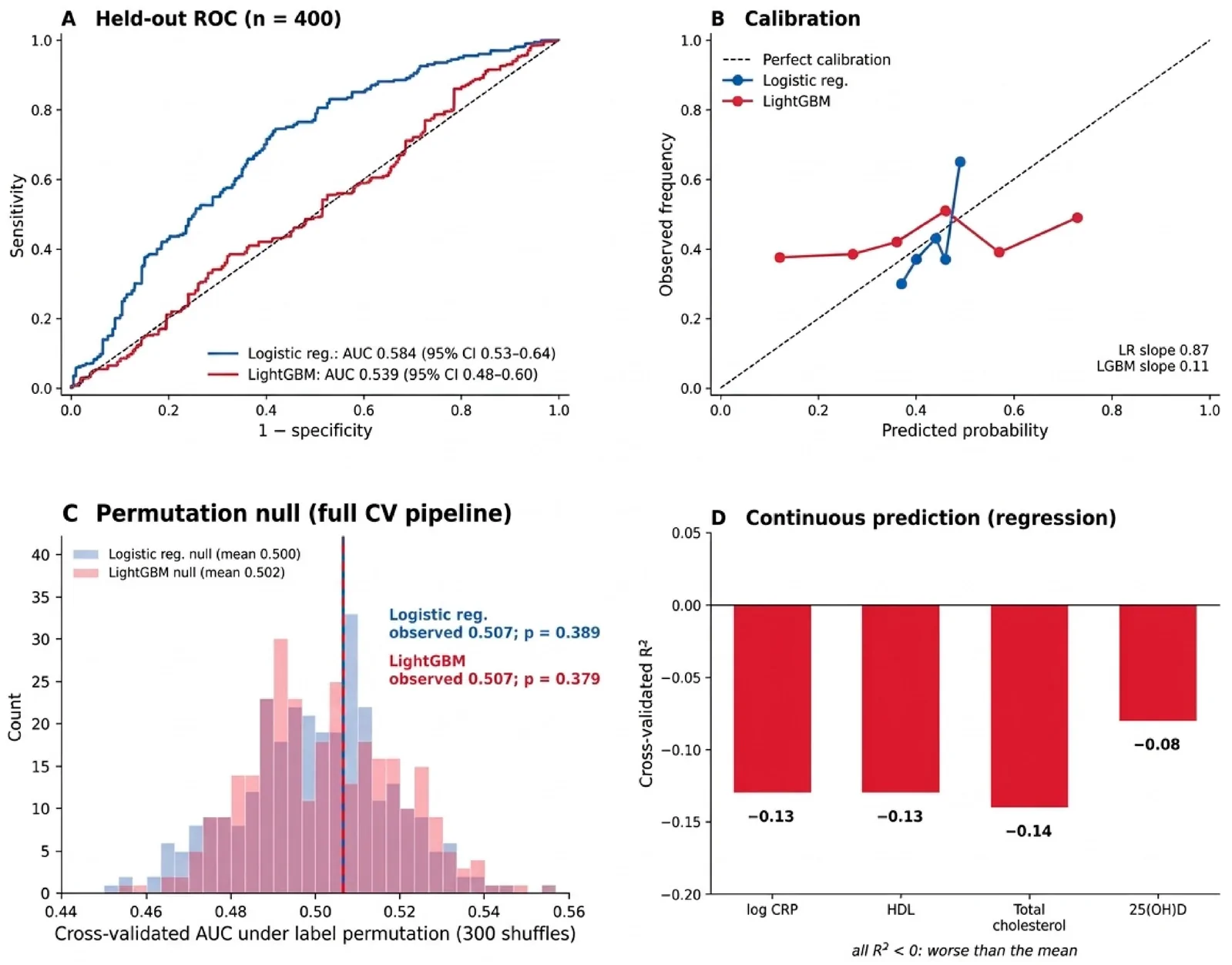
Held-out discrimination, calibration, permutation tests, and continuous outcomes. (**A**) Held-out ROC curves with bootstrap confidence intervals. (**B**) Calibration. (**C**) Permutation-null distributions of the pooled out-of-fold AUC for the complete cross-validated logistic-regression and LightGBM pipelines (300 permutations each), with observed values; neither is separable from chance. (**D**) Continuous-outcome regression: all cross-validated R^2^ are negative.

**Figure 4:**
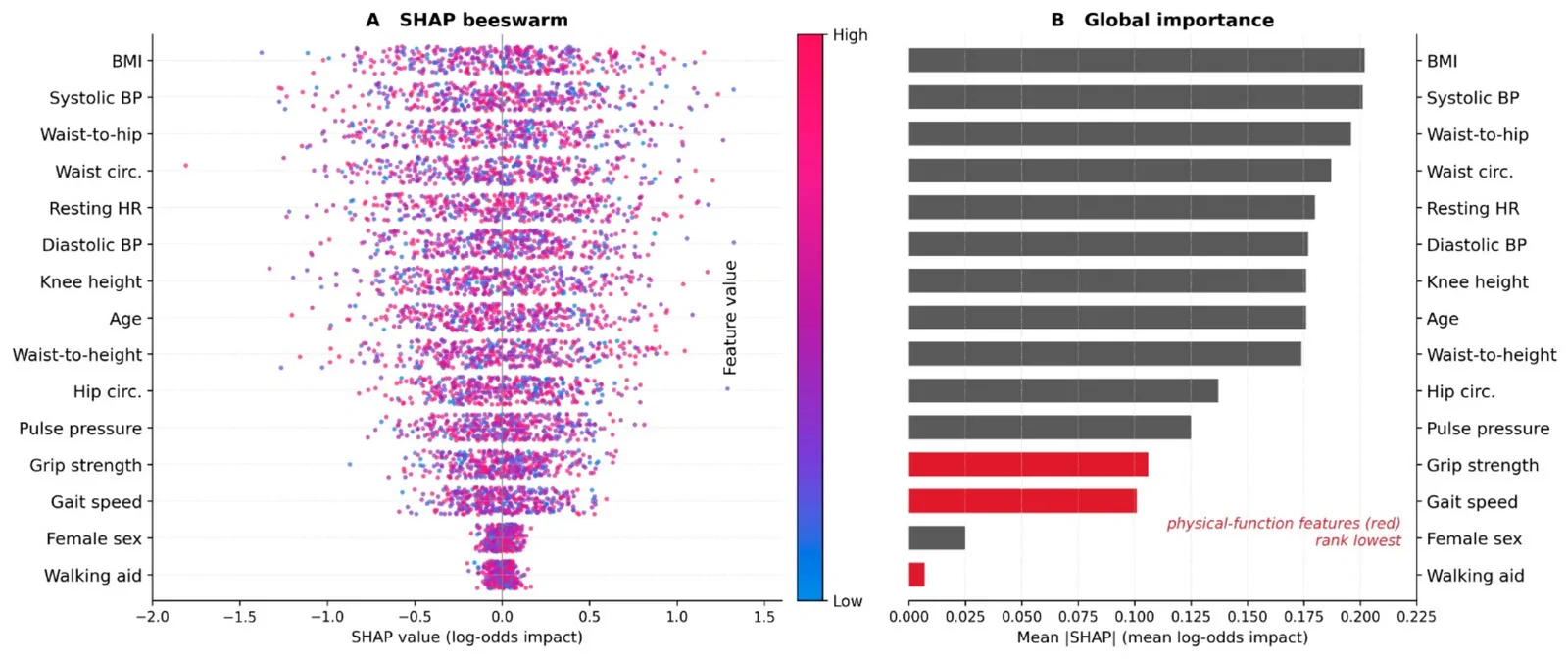
SHAP attributions for the flexible model. (**A**) Bee swarm and (**B**) global mean-|SHAP| importance. Importance is diffuse, and grip strength and gait speed rank below all anthropometric and vital-sign features. Given near-chance discrimination, these attributions largely reflect in-sample noise.

**Figure 5:**
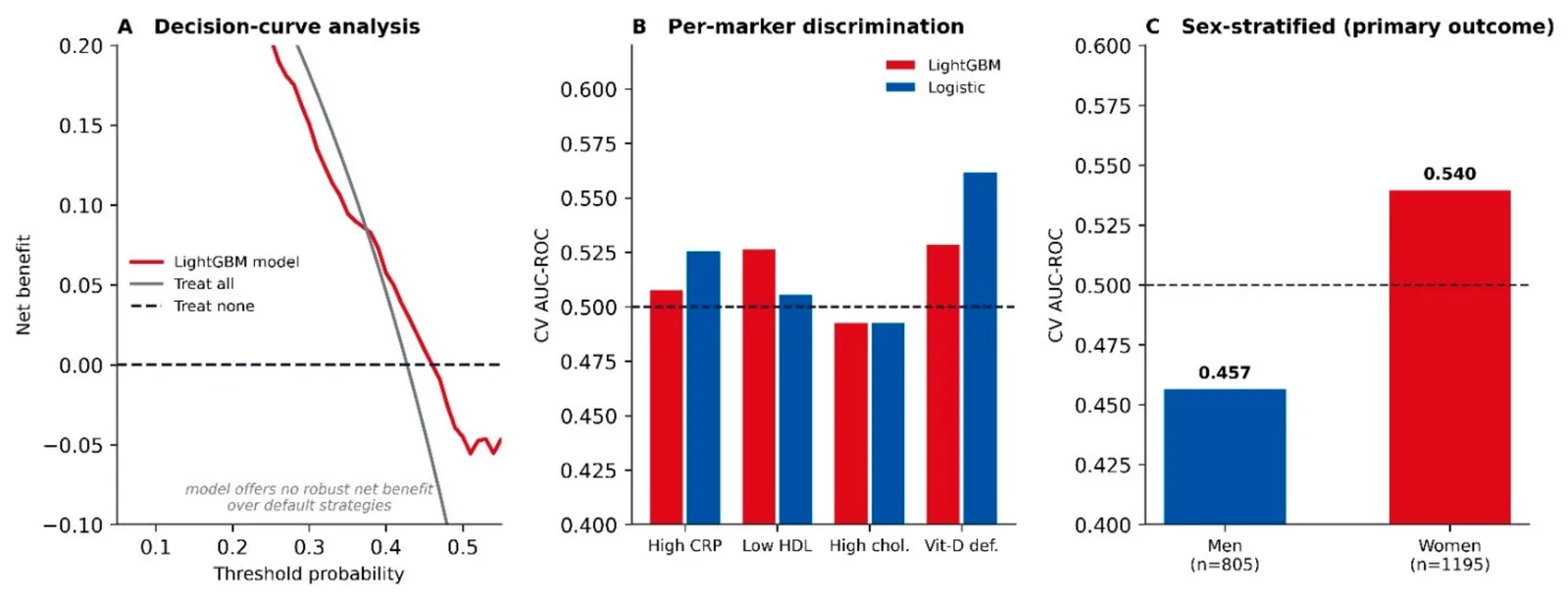
No clinical utility and no rescuing subgroup. (**A**) Decision-curve analysis (net benefit vs. threshold). (**B**) Per-marker cross-validated discrimination. (**C**) Sex-stratified discrimination of the primary outcome; the dashed horizontal line represents the chance level (AUC = 0.500).

**Table 1:** Summary of predictive performance metrics for the adverse blood-biomarker profile using needle-free measures.

Analysis	Metric	Result (Needle-Free → Blood-Biomarker Profile)
16-algorithm CV sweep	ROC AUC range	0.481—0.524 (all near chance)
Held-out test—LightGBM	AUC (95% CI)	0.539 (0.477–0.596); Brier 0.273; slope 0.107
Held-out test—logistic	AUC (95% CI)	0.584 (0.528–0.638); Brier 0.24; slope 1.892
Held-out test—logistic, 200 random 80/20 splits	Median AUC (2.5th–97.5th percentile)	0.518 (0.473–0.567); fixed-split permutation *p* = 0.007
Permutation tests (300), full CV pipeline	*p*-value	LR 0.389; LightGBM 0.379 (not separable from chance)
Continuous regression	CV R^2^ (4 markers)	−0.14 to −0.08 (all < 0)
Per-marker discrimination	CV AUC range	0.49–0.56
Sex-stratified	CV AUC	men 0.457; women 0.54
Decision-curve analysis	Net benefit	≈treat-all/treat-none (no utility)
Sensitivity analyses (Table 2)	CV AUC range	0.489–0.573 (none clinically useful)

Note: Results represent the mapping from 15 needle-free predictors to the blood-biomarker profile (*n* = 2000). ROC AUC: area under the receiver operating characteristic curve (0.50 = chance); CV: stratified 5-fold cross-validation; 95% CI: 800-sample bootstrap confidence interval; R^2^: coefficient of determination (negative values indicate performance worse than the sample mean); LightGBM: light gradient-boosting machine; LR: logistic regression. Held-out estimates come from a single stratified 80/20 partition of the same sample (internal validation). The adverse profile is defined as the presence of ≥2 abnormal markers (high CRP, low HDL, high total cholesterol, or vitamin D deficiency). All models were trained and validated using a leakage-controlled pipeline under TRIPOD+AI guidelines.

**Table 2:** Sensitivity analyses: cross-validated discrimination of the adverse blood-biomarker profile (primary outcome unless stated) by logistic regression (LR) and LightGBM.

Analysis	*n*	Prev.	LR AUC (95% CI)	LightGBM AUC (95% CI)
Reference: ≥2 of 4 abnormal markers	2000	42.8%	0.507 (0.482–0.533)	0.507 (0.482–0.531)
≥1 of 4 abnormal markers	2000	83.9%	0.533 (0.498–0.569)	0.516 (0.484–0.550)
≥3 of 4 abnormal markers	2000	10.8%	0.515 (0.473–0.556)	0.517 (0.476–0.560)
≥2 of 3 cardiometabolic markers (vitamin D excluded)	2000	28.0%	0.504 (0.475–0.530)	0.522 (0.493–0.551)
Any lipid abnormality (low HDL or high total cholesterol)	2000	57.0%	0.508 (0.481–0.532)	0.495 (0.467–0.521)
Without SMOTE	2000	42.8%	0.510 (0.485–0.537)	0.506 (0.481–0.529)
Positive survey weight, unweighted	1826	43.0%	0.533 (0.504–0.559)	0.518 (0.491–0.543)
Positive survey weight, weighted training and AUC	1826	45.3%	0.537 (0.477–0.587)	0.552 (0.506–0.597)
HbA1c < 6.5% (lower likelihood of lipid-lowering therapy)	1111	42.1%	0.505 (0.473–0.540)	0.514 (0.481–0.550)
No health insurance (less access to pharmacotherapy)	349	44.4%	0.573 (0.509–0.632)	0.539 (0.480–0.600)
Age ≥ 50 years	1857	43.3%	0.489 (0.460–0.515)	0.492 (0.464–0.517)
Extended needle-free predictor set	2000	42.8%	0.507 (0.481–0.535)	0.502 (0.476–0.528)

Note: All analyses use the 15 needle-free predictors, stratified 5-fold cross-validation (seed 42), the pooled out-of-fold AUC, and 1000-sample bootstrap 95% CIs. The extended set adds day of blood draw, locality size, health-insurance affiliation, and new versus follow-up cohort. SMOTE was applied within training folds except in the rows without SMOTE and with weighted training. Weighted analyses used the MHAS 2012 individual weight, and the prevalence shown is weighted. Mean AUCs over 10 repetitions of cross-validation differed from the values shown by ≤0.02. HbA1c: glycated hemoglobin (point of care), used only to define the stratum. Prev.: prevalence. These analyses were added during peer review and are post hoc.

## Data Availability

The data presented in this study are openly available in the Mexican Health and Aging Study (MHAS) repository. The original data presented in the study are openly available at https://mhasweb.org/. Restrictions apply to the availability of the 2012 biomarker and physical fitness data used in this specific analysis, which were collected from a subsample of participants. These data are available from the corresponding author upon reasonable request and with the permission of the MHAS Steering Committee.

## Abbreviations

The following abbreviations are used in this manuscript.

AI	artificial intelligence
AUC	area under the curve
BMI	body mass index
CI	confidence interval
CRP	C-reactive protein
CV	cross-validation
DNAm	DNA methylation
HbA1c	glycated hemoglobin
HDL	high-density lipoprotein
k-NN	k-nearest neighbors
LightGBM	light gradient-boosting machine
LR	logistic regression
MHAS	Mexican Health and Aging Study
ML	machine learning
PROBAST	prediction model risk-of-bias assessment tool
ROC	receiver operating characteristic
R^2^	coefficient of determination
SHAP	Shapley additive explanations
SMOTE	synthetic minority oversampling technique
TRIPOD	transparent reporting of a multivariable prediction model for individual prognosis or diagnosis
